# Obesity and Infertility: A Metabolic Assessment Strategy to Improve Pregnancy Rate

**Published:** 2020

**Authors:** Rachel Talia Bond, Alexandra Nachef, Catherine Adam, Marielle Couturier, Isaac-Jacques Kadoch, Louise Lapensée, Gilles Bleau, Ariane Godbout

**Affiliations:** 1- Endocrinology Division, Department of Medicine, Centre de Recherche du Centre Hospitalier de l'Université de Montréal (CRCHUM), Montreal, Quebec, Canada; 2- Clinique de Procréation Assistée du Centre Hospitalier de l'Université de Montréal (CHUM), Department of Obstetrics and Gynecology, Montreal, Quebec, Canada

**Keywords:** Assisted reproductive techniques, Infertility, Metabolic syndrome, Obesity, Vitamin D deficiency

## Abstract

**Background:**

The metabolic global approach is a multidisciplinary intervention for obese women before undergoing assisted reproductive techniques, with the goal of improving fertility and decreasing adverse pregnancy outcomes. The objective of this study was to evaluate the impact of the metabolic global approach on pregnancy rate.

**Methods:**

This retrospective cohort study included 127 women and was conducted at the Centre hospitalier de l’Université de Montréal fertility center. Eligibility included BMI at initial consultation of ≥30 *kg/m*^2^. Fertility treatments were considered when a weight loss of minimum 5% and normal metabolic indices were achieved. The p<0.05 was considered statistically significant.

**Results:**

Median baseline and last clinical assessment BMIs were 38.2 *kg/m*^2^ and 35.8 *kg/m*^2^ respectively (p<0.001), representing a median weight loss of 5.1%. At baseline, at least one metabolic parameter was abnormal in 66% of women. Total pregnancy rate was 53%. The majority of women (63%) who achieved pregnancy did so with weight loss and metabolic stabilization alone (11%) or combined with metformin (36%) and/or oral ovulation drugs (16%). Normal vitamin D (p<0.001) and triglyceride levels (p<0.05) as well as lower BMI after weight loss (p<0.05) were associated with an increased relative risk of pregnancy.

**Conclusion:**

Replete vitamin D status, weight loss of 5% and lower BMI as well as normal triglyceride level are significant and independent predictors of pregnancy in obese women presenting to our fertility center. The metabolic global approach is an effective program to detect metabolic abnormalities and improve obese women’s pregnancy rate.

## Introduction

The proportion of overweight or obese women in Canada has increased to 54% in 2014 (1). Several authors have demonstrated the impact of obesity on fertility. In obese women, each unit increase of Body Mass Index (BMI) is associated with a 5% decrease of ovulatory function (2). As well, higher categories of BMI are associated with lower rates of pregnancies and live birth rates (3) and higher risk of obstetrical complications such as gestational diabetes, hypertensive disorders, assisted delivery, caesarean section and neonatal morbidity (4).

Obesity represents a challenge in fertility clinics despite improvements in assisted reproductive techniques (ARTs). Poor outcome has been observed in obese women throughout fertility treatments. The success of ART is decreased in obese women due to lower ovarian response, poor quality oocytes (5, 6) and unfavorable endometrial changes (7). Despite ART, overweight women remain with a lower pregnancy rate and higher miscarriage rate compared to women with a BMI <25 *kg/m*^2^ (3, 8).

A weight loss of 5% to 10% coupled with lifestyle changes is associated with regulation of menstrual cycle, increased spontaneous ovulation and improved pregnancy rate (9, 10). Chavarro et al. showed a positive impact of weight loss on the results of fertility treatments, specifically by improving oocyte quality (11). This study had insufficient power to conclude on the impact of weight loss alone on pregnancy outcomes in obese women. This suggests, as reported in other studies, that maternal weight is not the only factor to address in order to improve pregnancy rate in these patients (12, 13).

Maternal obesity is associated with adverse metabolic complications including type 2 diabetes, hypertension, dyslipidemia and metabolic syndrome (14, 15). These comorbidities need to be screened prior to undergoing ART as they could affect female reproductive ability and increase risk of adverse pregnancy outcomes when left untreated (16).

Moreover, it has been demonstrated that obesity is associated with vitamin D deficiency (17). Vitamin D insufficiency has been suggested as a factor influencing female reproductive ability (18) and IVF outcomes (19). It remains unclear if vitamin D supplementation is associated with higher pregnancy rate after IVF (20, 21); therefore, further research is needed.

In order to assess maternal metabolic risk before undergoing ART, the metabolic global approach was developed at the Centre hospitalier de l’Université de Montréal (CHUM) in order to create a multidisciplinary intervention to assess and treat obese women presenting for ART. The aim of the present study was to evaluate this program's impact on pregnancy rate. This is the first study to evaluate a combined approach targeting obesity, metabolic syndrome and vitamin D status, prior to ART, on pregnancy rate.

## Methods

### Study design:

This single-center retrospective cohort study was approved by the ethics committee of the CHUM in conformity with the Declaration of Helsinki. Data was collected from patients’ medical records at the CHUM and affiliated ART centers. Upon presentation to the CHUM fertility center, all patients with BMI ≥30 *kg/m*^2^ at initial consultation, between October 2011 and February 2014, were included, resulting in 129 patients. Exclusion criteria included BMI <30 *kg/m*^2^, presence of a medical condition precluding conception, as well as having a medical chart with incomplete data, which excluded 2 patients. These criteria provided us with a sample size of 127 patients. All included patients underwent a 75 *g* oral glucose tolerance test (OGTT) and nutritional consultation prior to initiation of ART. Consultation with an endocrinologist and additional metabolic work-up were added if BMI was ≥35 *kg/m*^2^ or if clinically indicated after initial evaluation. This additional metabolic work-up included a lipid profile, liver function tests, serum 25(OH)-vitamin D level and blood pressure (BP) measurement. Throughout the process, the patients were followed as clinically indicated by a registered nutritionist for weight-loss counseling. They were advised to adhere to an energy-reduced balanced healthy diet with a caloric reduction of approximately 500 *kcal* compared to the calculated daily needs (According to the Institute of Medicine (IOM) recommendations based on age, adjusted weight and height) (22). Cardinal recommendations also included regular physical activity (Minimum of 150 *min/week* of moderate-to-vigorous intensity physical activity, divided in sessions of ≥10–15 *min* several times per week) (23). Based on a completed local survey of personal health practices and smoking status, instructions to improve lifestyle were given and regularly reviewed with the patients. Folic acid (5 *mg/d*) and vitamin D (1000 *IU/d*) supplements were prescribed to all subjects, following the recommendations for obese women (24, 25). Serum levels of 25(OH)-vitamin D were measured. Metformin was initiated if pre-diabetes or type 2 diabetes was diagnosed to achieve normal glycemic control.

Follow-up frequency was determined individually as clinically relevant. During follow-up, after a significant weight loss, oral ovulation induction drugs (Clomiphene citrate or letrozole) were used if clinically appropriate. IVF or inseminations were only initiated when women were metabolically stable, defined as weight loss of more than 5%, normal OGTT or controlled prediabetes/diabetes (A1c <7%), normal triglycerides level (<1.7 *mmol/L*, controlled BP (≤130/85 *mmHg*), normal liver function (AST/ALT within normal range), and adoption of a healthy lifestyle such as smoking cessation.

When fertility treatments were permitted, follow-up was made with the treating gynecologist. The protocols used have been previously described in literature reviews. Treatments were chosen according to the infertility diagnosis made by the specialist physician. As it was felt to be unethical to have a control group where obese patients are denied access to a multidisciplinary team with clinically appropriate metabolic assessment and intervention, there is no control group in this study.

### Exposure:

The aim of the study was to look at the association between metabolic parameters and fertility. The metabolic parameters assessed were glucose tolerance, dyslipidemia, liver function, hypertension and vitamin D status. Glucose tolerance was defined (26) as being normal (Fasting plasma glucose (FPG) ≤6.0 *mmol/L* or 106 *mg/dl* and/or a 2 *hr* plasma glucose ≤7.7 *mmol/L* or 136 *mg/dl* after a 75 *g* OGTT), prediabetes (FPG 6.1–6.9 *mmol/L* (110–124 *mg/dl*) and/or a 2 *hr* plasma glucose 7.8–11.0 *mmol/L* (141–198 *mg/dl*) after a 75 *g* OGTT and/or an A1c of 6.0–6.4%) or type 2 diabetes (FPG ≥7.0 *mmol/L* (126 *mg/dl*), a random plasma glucose value ≥11.1 *mmol/L* (200 *mg/dl*) or an A1c ≥6.5% on two different occasions). Vitamin D sufficiency was defined as serum levels of 75–150 *nmol/L* and vitamin D insufficiency and deficiency as serum levels of 50–74 *nmol/L* or <50 *nmol/L* (27, 28), respectively. Subjects were considered metabolically normal obese (MNO) if they had all normal metabolic indices or as metabolically abnormal obese (MAO) if at least one of the above parameters was not in the normal range at first assessment.

### Outcome measures:

Pregnancy was the primary outcome of interest. Pregnancy was confirmed by a positive β-hCG serum test and/or with first trimester ultrasound showing a positive fetal heart rate (FHR) at 7 weeks gestation by last menstrual period (LMP) dating. Biochemical pregnancy was defined as having a positive β-hCG serum test, but no embryo was visualized on ultrasound. Clinical pregnancy was defined by ultrasound showing a positive FHR at 7 weeks gestation. Infertility was defined for heterosexual couples as inability to conceive after 12 months of attempt.

### Statistical analyses:

Statistical analyses were performed using SPSS Statistics version 21. Continuous variables were summarized as mean±standard deviation (SD) or as median and interquartile range (IQR) if skewed. ANOVA, non-parametric Mann-Whitney U-test or Kruskal -Wallis test were used for continuous variables. The Wilcoxon signed-rank was used for paired data, and the Fishers’ exact test was used for categorical outcomes. Cumulative pregnancy rates over time were calculated for each group and subgroup using the Kaplan-Meier method. As occurrence of pregnancy was the dependent variable, the log-Poisson regression with the Generalized Estimating Equations procedure was used to estimate relative risks (RR) and adjusted relative risks (aRR) along with their 95% confidence intervals (95% CI); adjustment of relative risk was limited to 3 clinically and statistically (Univariate analysis) significant covariates. All p-values are two sided, and p<0.05 was considered statistically significant.

## Results

From October 2011 to February 2014, 129 obese women presenting to our fertility center were included in the program. Two patients were excluded from the study: one because of a newly diagnosed endometrial adenocarcinoma limiting conception and the other due to having no further desire of conception. Patients’ baseline characteristics are described in table 1.

**Table 1.** Baseline characteristics and final weight and BMI of participants

**Number of patients (n)**	127	
**Age at enrolment (y, M±SD)**	32.6±5.0	
**Number of prior live births (n, %)**				
0	60 (47)	
≥1	67 (53)	
**Ethnicity (n, %)**				
Caucasian	79 (62)	
Middle-East/North Africa	17 (13)	
Haitian Black	16 (13)	
Hispanic	2 (1.5)	
Asian	2 (1.5)	
Others	11 (9)	
**Infertility etiology (n, %)**				
PCOS	31 (24)	
Male factor	26 (21)	
Low ovarian reserve	30 (24)	
Anatomic (tubal or uterine)	14 (11)	
Single women/same sex couple	13 (10)	
Unexplained	13 (10)	
**Initial weight (*kg*; median [IQR])**	102.5	[93.6; 113.6]
**Final weight (*kg*; median [IQR])**	97.3	[86.4; 108.2]^*^
**Initial BMI (*kg/m*^2^; median [IQR])**	38.2	[35.1; 41.9]
**Final BMI (*kg/m*^2^; median [IQR])**	35.8	[33.3; 39.3] ^*^
**Current smoking (n, %)**	12 (9.7)	

* final <initial (p<0.001). PCOS: polycystic ovary syndrome; BMI: body mass index. PCOS diagnosis was made according to the Rotterdam criteria and exclusion of other condition mimicking PCOS. Low ovarian reserve diagnosis was made with calculation of the antral follicle count (AFC) on transvaginal ultra-sound on day 2 to 5 of a menstrual cycle (spontaneous or induced with medroxyprogesterone acetate) and low AMH level (<1.4 *ng/ml*)

After a mean follow-up period of 7.5 months, the median weight loss for the entire cohort was 5.2 kg with a median BMI of 35.8 *kg/m*^2^ at the end of the study (p<0.001) (Table 2). Half of the patients succeeded in reducing their body weight by more than 5%, which was the minimum weight loss targeted at first consultation.

**Table 2.** Comparisons between metabolically normal obese and metabolically abnormal obese patients^a^

	**Metabolically Normal Obese (MNO)**	**Metabolically Abnormal Obese (MAO)**			**p-value**

		**Whole MAO group**	**Corrected post-intervention**	**Not corrected post-intervention**	
**Number of patients (n)^b^**	42	81	25	29	
**Age (y) (mean±SD)**	32.1±4.9	32.8±5.0	32.2±5.2	33.4±4.7	0.490
**Initial weight (*kg*) (median [IQR])^b^**	100.2 (20.8)	104.1 (23.2)	104.0 (23.4)	107.3 (25.8)	0.410
**Final weight (*kg*) (median [IQR])**	91.8 (21.4)	98.2 (24.2)	94.5 (23.1)	99.6 (26.4)	0.242
**Weight loss (%) (median [IQR])**	5.2 (7.8)	4.8 (7.2)	7.0 (9.1)	4.8 (5.9)	0.157
**Initial BMI (*kg/m*^2^) (median [IQR])**	38.5 (6.2)	38.2 (7.6)	38.8 (9.2)	38.8 (8.3)	0.610
**Final BMI (*kg/m*^2^) (median [IQR])**	35.9 (6.5)	35.8 (6.4)	36.5 (8.4)	37.1 (8.0)	0.830
**Pregnancy rate/women**	20/42 (48%)	38/81 (47%)	12/25 (48%)	12/29 (41%)	0.494
**Biochemical pregnancies (+hCG) (n;%)**	24 (57%)	43 (53%)	15 (60%)	12 (41%)	0.340
+hCG after ART (n)	11	14	4	3	0.150
+hCG without ART (n)	13	29	11	9	0.320
**Miscarriages (n) (% pregnant women)**	7 (35%)	16 (42%)	6	5	0.317

a: Comparisons: MNO versus MAO whole group; MAO corrected versus not corrected.

b: Data not available in 4 patients; data available in 54 patients for MAO corrected and not corrected combined. SD: Standard deviation; IQR: Interquartile range; BMI: Body mass index

Upon first metabolic evaluation, 81/123 patients (66%) were classified as MAO (Table 2, full data not available for 4 patients). Of the patients for whom data was available, prediabetes/type 2 diabetes (50/121 (41%)) and hypertriglyceridemia (33/113 (29%)) were the most common newly diagnosed metabolic anomalies. A total of 84/127 (66%) women were treated with metformin in vast majority after prediabetes or type 2 diabetes was diagnosed. Through participation in the program, further laboratory work-up showed normalization of metabolic parameters in 25/81 (31%) of MAO patients who were subsequently classified as metabolically corrected patients. It was also noted that 56/77 (73%) of the patients tested for vitamin D status were found to be vitamin D insufficient/deficient.

During follow-up, 67/127 (53%) biochemical and clinical pregnancies were recorded in 58 women (Table 3). Forty-two (42/67, 63%) of the pregnancies were achieved without ART, either with weight loss and metabolic stabilization alone (11%) or combined with metformin (36%), and/or oral ovulation drugs such as clomiphene citrate (15%) or letrozole (2%). ART was used in 43/127 (34%) of patients, of whom 22/43 achieved pregnancy either with IUI (n=10), donor insemination (DI) (n=6) or IVF (n=6). The main outcome of this study was pregnancy (Table 3). Statistically significant differences between pregnant and non-pregnant women were observed for hypertriglyceridemia (aRR 1.91, 95% CI 1.01, 3.59), final BMI (aRR 0.95, 95% CI 0.90, 0.99) and normal vitamin D (aRR 2.74, 95% CI 1.67, 4.49) status, with lower BMI, normal triglycerides and sufficient vitamin D level all favoring pregnancy. At the end of the study, each unit of BMI lost on regression analysis was associated with a 5% increase in pregnancy rate (p=0.045).

**Table 3.** Comparisons of metabolic parameters between pregnant and non pregnant patients

	****Pregnancy****	****No pregnancy****	****Relative risk****
**Continuous variables (n=127)**	67/127	60/127	
Age (y)^i^	32.0±4.7	33.0±5.1	0.97 (0.94–1.02)
Initial body weight (*kg*)^ii^	104.0 (18.3)	101.8 (23.2)	0.99 (0.98–1.01)
Final body weight (*kg*)^ii^	95.2 (20.1)	98.2 (23.1)	0.99 (0.98–1.01)
Weight loss (%)^ii^	5.3 (9.2)	4.6 (6.7)	1.01 (0.97–1.02)
Initial BMI (*kg/m*^2^)^ii^	38.0 (6.2)	38.3 (8.1)	0.99 (0.96–1.03)
Final BMI (*kg/m*^2^)^ii^	35.6 (5.8)	36.3 (9.0)	0.95 (0.90–0.99)^a*^
Fat mass (%)^i^ (n=121)	52.0±4.5	52.1±4.6	0.99 (0.95–1.04)
**Dichotomous variables^iii^**						
Metabolically normal obese (n=42/123)	20	22	1.04 (0.70–1.55)^2^
Metabolically abnormal obese corrected (n=25/54)	12	13	1.16 (0.64–2.11)^2^
Prediabetes (n=36/121)	15	21	1.03 (0.45–2.36)^3^
Type 2 diabetes (n=14/121)	5	9	1.36 (0.64–2.77)^3^
Smoking (n=12/124)	6	6	0.89 (0.49–1.63)^3^
Hypertension (n=14/105)	7	7	0.86 (0.48–1.52)^3^
Abnormal liver function (n=13/70)	7	6	0.82 (0.45–1.46)^3^
Hypertriglyceridemia (n=33/113)^4^	13	20	1.91 (1.01–3.59)^a3*^
Normal 25(OH)-vitamin D level (n= 21/77)^5^	13	8	2.74 (1.67–4.49) ^a2**^

Results are presented as

i =mean ± SD,

ii =median and [IQR], or

iii =n;

*: p<0.05;

**: p<0.001

a: Adjusted relative risk - the 3 variables included in the model for aRR are final BMI, hypertriglyceridemia and normal vitamin D level.

2: not pregnant;

3: pregnant as reference for the variables;

4: For normotriglyceridemia (n= 80/113; 40 pregnant);

5: For abnormal 25(OH)-vitamin D level (n= 56/77; 19 pregnant).

BMI: Body mass index; SD: Standard deviation; IQR: Interquartile range; aRR: Adjusted relative risk

The group of MNO was compared to the group of MAO patients (Table 2). Within each group, the final weight was significantly lower than the initial weight (p<0.001, analysis on paired data); however, there was no significant difference between groups for the variables examined.

**Figure 1.**
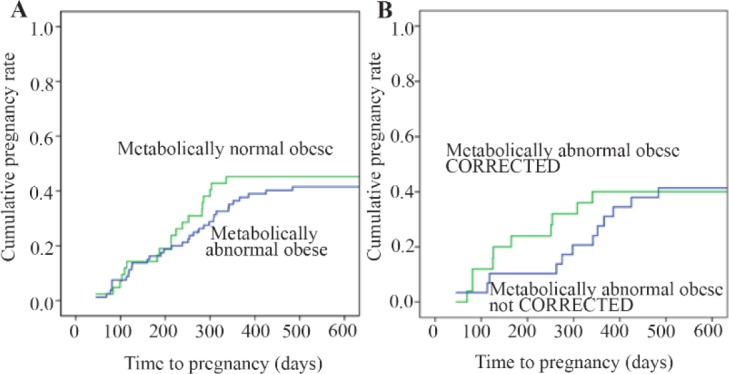
Cumulative pregnancy rates according to metabolic status (Kaplan-Meier analysis)

The cumulative rates of pregnancy were calculated for the MNO and MAO groups and subgroups and the Kaplan-Meier curves are presented in figure 1. While the patients in the MNO group tended to conceive more rapidly than the MAO patients, the difference was not statistically significant (Log-rank test: 0.227, p=0.739).

## Discussion

The metabolic global approach offers a comprehensive evaluation of the metabolic profile of obese women presenting to our fertility center before undergoing fertility treatments. To our knowledge, this is the first study to examine the impact of a comprehensive metabolic approach on pregnancy rate. Two-thirds of patients in our cohort had at least one metabolic abnormality at initial evaluation. Women who became pregnant were significantly more likely to have a normal triglyceride level, lower BMI and replete vitamin D status, compared to women who did not become pregnant, indicating the importance of these specific metabolic parameters in fertility.

Pregnancy rates were similar in the MAO, MNO and corrected-MAO groups. The inconclusive associations found may be due to the short duration of follow-up, the small sample size and to the fact that all women received similar care and lifestyle advice to favor pregnancy.

The relationship between weight loss and fertility outcomes remains inconclusive. Clark et al. reported an improved pregnancy rate (77%) and live birth rate (67%) in infertile obese women who completed a lifestyle intervention program and lost 10% of their baseline weight, compared to those who lost only 1% of their body weight (12). More recently, Kort et al. showed higher conception (88% *vs*. 54%) and live birth rates (71% *vs*. 37%) in overweight infertile women (BMI ≥25 *kg/m*^2^) achieving weight loss compared to those who did not (29). However, a recent randomized controlled trial (RCT) did not find that weight loss in obese women prior to undergoing IVF improved live birth rate compared to women who had no weight loss intervention prior to ART (30).

It has been demonstrated that for the same BMI, a large spectrum of different metabolic profiles are possible (31). It was found that a normal triglyceride level was associated with a better pregnancy rate in our cohort. Weight loss can improve metabolic parameters, including triglyceride level (32), perhaps accounting for the positive association between weight loss and fertility in some studies. It has been demonstrated that hypertriglyceridemia correlates with insulin-resistance and higher level of inflammatory cytokines, parameters that have been postulated to interfere with implantation and conception (33–36). Our results suggest that triglyceride levels should be monitored at baseline and that women with hypertriglyceridemia should be encouraged to normalize their lipids profile prior to conception in order to improve their chance of conception.

In our cohort, obese women presenting to our clinic with sufficient vitamin D levels were 2.7 times more likely to achieve pregnancy than the insufficient/deficient group. This finding reinforces recent research, which shows that women with a higher 25(OH)-vitamin D serum level had better clinical pregnancy rates compared to those with vitamin D deficiency/insufficiency (37). Proposed mechanisms for the role of vitamin D in fertility include: vitamin D as a marker of socio-economic factors (Diet, obesity, exposure to sunlight, sedentary lifestyle), as being implicated in implantation (38), gonadal steroidogenesis (39, 40) and in increasing anti-mullerian hormone levels (41).

Limitations of the present study include its real-world design and the absence of a control group. Our approach was not compared to a standard protocol, as for ethical reasons, all women with a BMI ≥30 *kg/m*^2^ were included in this program. Comparing our outcomes to those followed at our clinic prior to program implantation was difficult, due to limited data availability. Also, missing data reduced sample size for some analyses, most notably for vitamin D levels after supplementation. Formal information on socio-economic status as well as a detailed history of diet and on physical activity was not available. Despite the above limitations, a statistically significant difference was noted between pregnant and non-pregnant groups with respect to vitamin D status, triglycerides level and BMI. These findings suggest that the metabolic global approach allows for establishment of more specific metabolic goals prior to undergoing fertility treatments. Therefore, focusing on correcting specific underlying metabolic abnormalities, rather that exact weight or BMI per se, has the potential to identify women at high risk of having difficulty conceiving and high-risk pregnancies and give them tangible goals to attain pre-treatment, limiting potential discriminatory measures focusing solely on weight.

## Conclusion

Metabolic abnormalities are frequent and the metabolic global approach is an effective way to identify these abnormalities in obese women. Replete vitamin D status, lower BMI and normal triglyceride level at baseline are significant and independent predictors of pregnancy. A prospective study including a larger cohort is needed to evaluate if adequate vitamin D supplementation as well as correction of abnormal triglyceride levels increases pregnancy rate in obese women presenting for fertility treatments. It seems that metabolic global approach has promise to improve pregnancy rates in obese women and possibly reduce the need for further fertility treatments as well as improve the safety of these much-desired pregnancies.
